# Rethinking of the Roles of Endophyte Symbiosis and Mycotoxin in *Oxytropis* Plants

**DOI:** 10.3390/jof7050400

**Published:** 2021-05-20

**Authors:** Huirui Guan, Xin Liu, Luis A. J. Mur, Yanping Fu, Yahui Wei, Jing Wang, Wei He

**Affiliations:** 1 Key Laboratory of Resource Biology and Biotechnology in Western China, Department of Life Sciences, Northwest University, Xi’an 710069, China; guanhuirui@163.com (H.G.); fuyanping@nwu.edu.cn (Y.F.); weiyahui@nwu.edu.cn (Y.W.); 2 Shaanxi Institute for Food and Drug, Xi’an 710065, China; liuxin_bio@sina.com; 3 Institute of Biology, Environmental and Rural Science, Aberystwyth University, Aberystwyth SY23 3FL, UK; lum@aber.ac.uk; 4 Key Laboratory of Grassland Resources of Ministry of Education, Inner Mongolia Agricultural University, Hohhot 010018, China; wangjing__3005@126.com; 5 Resources and Environment, College of Grassland, Inner Mongolia Agricultural University, Hohhot 010018, China

**Keywords:** *Oxytropis*, *Alternaria* sect. *Undifilum*, locoweed, symbiosis, swainsonine

## Abstract

Plants in the *Oxytropis* genus can live with the endophytic fungi *Alternaria* sect. *Undifilum*. Swainsonine, the mycotoxin produced by the endophyte render the host plant toxic and this has been detrimental to grazing livestock in both China and U.S.A. Despite previous efforts, many questions remain to be solved, such as the transmission mode and life cycle of host–endophyte symbiont, the biosynthesis pathway of swainsonine, and in particular the ecological role and evolution of such symbiosis. In this review, we compile the literature to synthesize ideas on the diversity of the symbiosis and propagation of the endophyte. We further compare the previous work from both *Alternaria* sect. *Undifilum* and other swainsonine producing fungi to orchestrate a more comprehensive biosynthesis pathway of swainsonine. We also connect swainsonine biosynthesis pathway with that of its precursor, lysine, and link this to a potential role in modulating plant stress response. Based on this we hypothesize that this host–endophyte co-evolution originated from the needs for host plant to adapt for stress. Validation of this hypothesis will depend on future research on endophytic symbiosis in *Oxytropis* and help in better understanding the roles of plant–endophyte symbiosis in non-Poaceae species.

## 1. Introduction

Locoweeds are forbs from the *Oxytropis* and *Astragalus* genus that are poisonous to grazing animals. The name (“loco” means crazy in Spanish) is historical as the consumption of locoweeds was observed to cause abnormal behavior in sheep and horses on rangelands of the U.S.A. [1,2,3]. Later on, several studies determined that the main toxic agent in locoweeds is swainsonine, an indolizidine alkaloid, which inhibits alpha-mannosidase II and causes neurotoxic symptoms and other pathogenic changes in animals [4,5,6,7]. Currently, locoweeds are the most problematic weeds on the rangelands in the U.S.A. and China [8,9]. An outbreak of locoweeds not only causes losses to the livestock industry, but also creates ecological problems because they can easily become dominant in rangeland plant communities [10,11,12]. This is particularly true in China as overstocking has been increasing over years due to the demand for meat consumption. Despite of extensive research into locoweeds focusing on biological, ecological, and toxicological aspects, currently there is no effective way to prevent locoweeds outbreaks.

Toxins in poisonous plants are not always produced by the plants themselves. Secondary metabolites caused animal poisoning by *Lolium perenne* aroused the research interests in plant–endophyte symbiosis. Endophyte symbiosis was previously considered to be mutualistic. This arose from early studies carried out in Poaceae, in which the symbionts formed with *Epichloë* (anamorph *Neotyphodium*) species produce toxic alkaloids and help the hosts deter herbivores and increase stress tolerance [13,14,15]. Numerous studies have focused on understanding the production and biosynthesis of swainsonine in locoweeds. Although initially recognized as a plant derived toxin, swainsonine was proved to be produced by endophytic fungi in locoweeds [16]. Different varieties of swainsonine-producing endophytes were isolated from locoweeds in the U.S.A. and China, and phylogenetic studies showed that they all belong to a redefined lineage as *Alternaria* sect. *Undifilum* [17,18,19]. The symbiosis between locoweeds and *A*. sect. *Undifilum* were considered most likely to be commensal (reviewed by Creamer and Baucom [20]), as despite toxins are produced, locoweeds do not have obvious herbivore deterrence to insects and grazing animals. In addition, the symbiosis also does not provide remarkable stress tolerance or growth promoting effects to the hosts. However, recent studies showed that *A*. sect. *Undifilum* might help their locoweed hosts against pathogens [21], or regulate the endophytic fungi assemblage [22]. Therefore, the ecological role of locoweed symbiosis awaits further investigation.

In this paper, we reviewed the classic studies of locoweeds to consider the latest development in our understanding in the relationships between host, endophyte, and swainsonine in locoweeds. We synthesized and compiled previous efforts with a focus to understand the diversity, and evolutionary and ecological significance of such symbiosis. As most locoweeds in China are *Oxytropis* species, and there have been many studies from the U.S.A. focusing on *Astragalus* locoweeds, we have particularly emphasized on research in *Oxytropis* locoweeds. This will also bring to an international audience locoweed papers that have been published in Chinese.

### 1.1. Distribution, Diversity, and Abundance of Oxytropis spp. and Alternaria sect. Undifilum

*Oxytropis* species are perennial forbs or semi-shrubs which belong to Fabaceae. There are approximately 310 *Oxytropis* species worldwide, mainly in Eurasia and America, within which 133 species (74 endemic) were documented in China [23]. Common *Oxytropis* locoweeds include *O. sericea* and *O. lambertii* in the U.S.A., which spread extensively in rangelands in western America, and causes animal poisoning in grazing livestock [24]. In China, *Oxytropis* locoweeds are more diverse, with the common ones being *O. glabra*, *O. ochrocephala*, *O. kansuensis*, *O. glacialis*, *O. sericopetata*, *O. deflexa*, *O. hirta*, *O. falcate,* etc. (Table 1). Those species can be found in all the four types of grasslands in China spanning Xinjiang, Gansu, Ningxia, Qinghai, Tibet, and Inner Mongolia (representing meadow steppe, typical steppe, desert steppe, and alpine steppe terrains [25]).

**Figure 1:**
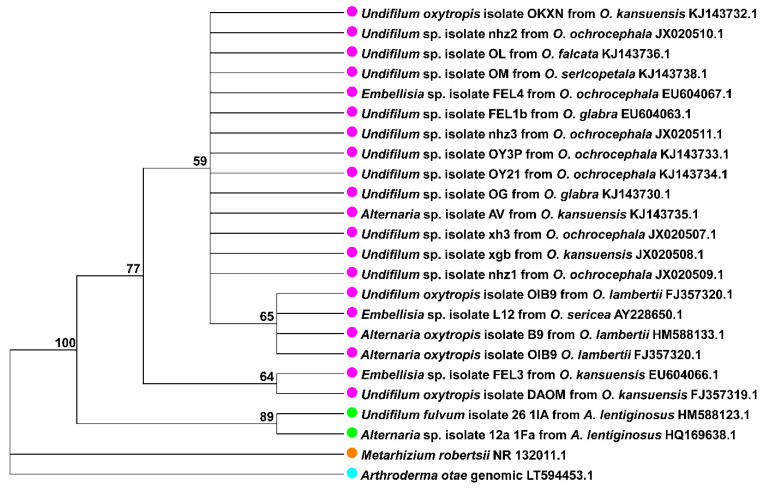
Maximum Likelihood phylogenetic tree constructed based on ITS sequences of 24 strains representing the swainsonine producing endophytes. The purple dots indicate fungi were isolated from the *Oxytropis* genus, and the green ones indicate the *Astragalus* genus. *Metarhizium robertsii* and *Arthroderma otae*, which also produce swainsonine, were used as the outgroup and were indicated in blue and orange, respectively. Bootstrap (1000 replicates) values were displayed.

Until recently, the taxonomic classification of the swainsonine producing endophytes in *Oxytropis* locoweeds was ambiguous. Braun et al. first isolated an endophyte from three locoweed populations in New Mexico and named it *Alternaria* sp. (Pleosporaceae) according to their morphological characteristics but rectified it to *Embellisia* sp. [19]. Wang et al. isolated a strain from *O. kansuensis* in China and found it to be similar to the one described by Braun, and named it *Embellisia oxytropis* [31]. In 2009, Pryor et al. combined *Embellisia* sp. and *Helminthosporium bornmuellerii* into *Undifilum* sp. [18]. In 2013, Woudenberg et al. constructed a phylogenetic tree across three loci (*GAPDH, RPB2* and *TEF1*), and moved *Undifilum* to a section of *Alternaria* [17]. Therefore, *U. oxytropis*, the most widely used name for the endophyte in *Oxytropis* locoweeds, was changed to *Alternaria* sect. *Undifilum* and this designation had been used thereafter (Table 2). The genetic variation of the *A.* sect. *Undifilum* conspecies is shown in Figure 1.

**Figure 2:**
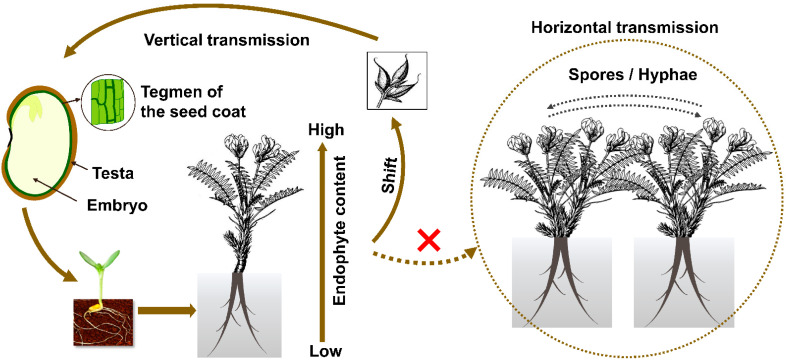
Model of transmission in *Oxytropis* locoweeds. Dotted line and the cross mark in red indicated horizontal transmission has not been found in the field for such symbiosis.

Endophyte colonization was found in various parts of the hosts in *Oxytropis*, and particularly in leaves and stems compared to roots during vegetative growth [37]. Eight *Embellisia* spp. were isolated from 11 locoweed populations in the U.S.A. The infection rates range were between 100% and 93.1% in flowers and seeds, and between 72.9% and 97.2% from leaves and stems [19]. During host reproduction, the endophyte is enriched in flowers, and this aids next generation transmission generation via the seed. Within the seed the endophyte is localized to the inside the seed coat (testa), and specifically in the endothelium parenchyma and aleurone layer [42]. No endophytes have been reported within the embryo and endosperm. Thus, the endophyte is transmitted through maternal material only, presumably from the integument in the ovary. Outside the seed, mycelium has been found between the intercellular spaces, and is most abundant in meristem rather than mature tissues [43,44]. This might be due to an efficiency in utilizing xylose and other polysaccharides [45]. In this context, it is also worth noticing that endophyte levels decrease in underground compared to aboveground tissues [46] (Figure 2). Therefore, it was speculated that locoweed endophytes may prefer to live in tissues with stronger photosynthetic activity and higher lysine levels [34], similar to the evidence demonstrated in grass and other plant species [47,48]. It is possible that the endophyte may preferentially infect certain tissue types or are distributed in a gradient as the plant grows from a seed to maturity plant.

Swainsonine is produced only by *A*. sect. *Undifilum* conspecies and many studies have aimed to identify biotic and abiotic factors that influence swainsonine production in the hosts. To address this, it is crucial to have accurate and effective detection methods of locoweed endophytes, both qualitatively and quantitatively. For qualitative detection, traditional approaches are based on plant culturing followed by fungal isolation from aseptic plant tissues [49,50] and remain effective for the morphological and molecular identification of the abundant and culturable endophyte taxon. Endophytes, including *A*. sect. *Undifilum* could be successfully cultured from plant leaf material from locoweeds [21]. This strategy can be supplemented by microscopic approaches to observe endophyte *in planta.* Endophytes can be viewed by aniline blue staining or electron microscopy [51]. Reyna et al. [44] later used scanning electron microscopy (SEM) and transmission electron microscopy (TEM) to localize and characterize the growth patterns of the fungus *Undifilum oxytropis* in *O. lambertii* and *O. sericea*. In their work, green fluorescence protein (GFP) transformed fungal strains were also successfully used to observe the colonization. Alternatively, endophytes could be cultured from plant material and PCR were employed for further identification of *A*. sect. *Undifilum*. For example, the ribosomal internal transcribed spacer (ITS) and β-tubulin were used as targets for amplification and identification *A*. sect. *Undifilum* endophyte in *O. sericea* and *O. lambertii* [19].

However, as many fungi could not be successfully cultured in vitro, PCR-based molecular identification without fungal culture has become more popular in laboratories. Gene specific primers can amplify regions targeting a specific fungus. This was first used successfully in *Festuca* by Doss et al. [52]. Based on the ITS region, which was the basis of locoweed endophyte specific primers OR1/ITS5 [27], Cook et al. developed a quantitative read-time (qPCR) method for the determination of the relative amount of endophytes and tested the method across three locoweeds in U.S.A [35] with a detection limit of 0.2 pg/ng (endophyte/plant DNA). This method has been used effectively in many locoweed species in both U.S.A. and China. A newly designed primer set, Omtssu F/Omtssu R was also tested and compared with OR1/ITS5 showed higher specificity and only amplified from locoweed endophyte DNA [53]. In our group, qPCR testing 19 *Oxytropis* species showed that the OR1/ITS5 had specificity in 17 species (data not shown) but not in *O. ochrocephala* and *O. kansuensis*, two morphologically close species. In *O. ochrocephala* OR1/ITS5 annealed to a chloroplast sequence with similar size, which will skew the data [54]. Thus, we suggest that using all the available qPCR methods after validation in the *Oxytropis* species being tested to avoid a lack of specificity. New primers could be developed based on genes on the swainsonine biosynthesis pathway (as in the *SwnK* cluster, see below) [55]. Alternatively, high-throughput sequencing could offer another way of measuring the relative abundance of locoweed endophyte based on distinguishing reads and counting reads hits. The full genome of *A*. sect. *Undifilum* has been studied that could help in providing a reference genome in amplicon sequencing.

### 1.2. Propagation and Reproduction of A. sect. Undifilum

Studies of the lifestyle of plant endophytes were greatly facilitated by the advance of microscopy. In locoweeds studied so far in U.S. and China, the precise location of *A.* sect. *Undifilum* within leaves and petioles is vascular bundle and pith portion, respectively. The hyphae live within cell walls but do not penetrate host cells, and no haustoria were observed. This suggests that *A.* sect. *Undifilum* is not pathogenic but most likely commensal because there are neither marked advantages when this symbiosis is formed comparing with the endophyte free plants [20,43].

Rodriguez and coworkers [56] classified endophytes into four groups based on their transmission and ecological interactions. Class 1 endophytes are those that infect a narrow host range, are generally vertically transmitted, and produce mycotoxins. Grass endophytes *Epichloe festuca* and *Neotyphodium* sp. fit within that grouping. Class 2 endophytes have a broad host range and are vertically and horizontally transmitted. Class 2 endophytes include *Phoma, Colletotrichum* sp., *Fusarium* sp., and *Curvularia* sp. Class 3 and Class 4 endophytes infect broad host ranges, are horizontally transmitted, and infect shoots and roots, respectively [20]. Unlike many other saprophytic endophytes that can generate spores, conidia of *A.* sect. *Undifilum* were never observed in *Oxytropis* plants in the past. *A.* sect. *Undifilum* do not generate gamobia with stroma and are vertically transmitted via seeds (Figure 2). The vertical transmission rate is usually not 100%, indicating the loss of infection through generations [32,57]. Horizontal transmission has never been recorded in the field for the last 20 years, and therefore *A.* sect. *Undifilum* are presumed to be Class I endophytes.

In the laboratory surface-sterilized seeds following seed coat removal could be inoculated with an endophyte culture, but conidia from *A.* sect. *Undifilum* produced in culture do not infect plants. This could be attributed to that the plant does not recognize the fungus as a pathogen, nor does the fungus act like a pathogen, or puncture cell walls. This contrasts with the pathogenic *A. astragali* (*A. gansuense* comb. nov.) isolated from standing milkvetch (*Astragalus adsurgens*) which cause yellow stunt and root rot [53]. The different life history suggests that swainsonine producing *A.* sect. *Undifilum* in *Oxytropis* plants is not a pathogen. Recently, some exploratory work from Li’s group in Lanzhou University showed evidence of some previously unidentified life strategy of *A.* sect. *Undifilum* [58]. Firstly, in *O. glabra* and *O. ochrocepala*, hyphae grew out from petiole that was mechanically wounded, and the hyphae covered leaflets later in all out of the 26 plant leaves. Secondly, spores were observed from the plant stem cutting tissue cultured on plates. Nevertheless, whether those results could represent an unidentified phenomenon in the field is still unknown.

Beyond such board classifications, it is difficult to explain why the *Oxytropis* genus contain both toxic and non-toxic species. It is not difficult to understand that toxic and non-toxic accessions/landraces/individuals were found in the same species, such as in *O. lambertii* [36], because random loss of the endophyte during transmission occurs, as we discussed above. However, considering the speciation event under the genus, several species were fixed with no symbiotic *A.* sect. *Undifilum* whereas the other toxic species do. Indeed, toxin producing and non-producing species can be located in the same geographical area (personal observation). Since no vertical transmission was observed, naturally the non-toxic species would not be able to acquire such symbiosis and become toxic. This must indicate that in the non-toxic species, the endophyte was lost due to either selection in the common ancestor or genetic drift happened in parallel. In either case, genetic variation at certain loci might have significant impact on the symbiotic colonization. Nonetheless, it could not be ruled out that *A.* sect. *Undifilum* used to be saprophytic in evolution. On the other hand, whether *A.* sect. *Undifilum* is present in the soil remain unambiguous. Lu et al. [21] isolated several fungi from the soil locoweeds grew, and although those fungi produce swainsonine they did not belong to *A.* sect. *Undifilum.* It would be more precise to confirm the presence or absence of *A.* sect. *Undifilum* in the rhizosphere soil of *Oxytropis* plants using metagenomics or amplicon sequencing.

### 1.3. The Endophyte and the Biosynthesis of Swainsonine

Studies indicate that swainsonine is produced by the *A.* sect. *Undifilum* endophytes rather than the host plants of *Oxytropis* [19,57]. Toxic *Oxytropis* species all contain *A.* sect. *Undifilum* conspecies, and in vitro culture of those *A.* sect. *Undifilum* was able to produce swainsonine [19,27,30,57]. Swainsonine could not be detected (nil or below 0.001% *w*/*w*) in *Oxytropis* plants from seeds with the seed coat removed or whole seeds treated with fungicide [42,59]. In line with this, the aseptic *Oxytropis* seeds, which do not contain detectable swainsonine, when inoculated with *A.* sect. *Undifilum*, produced swainsonine (>0.01% *w*/*w*) [59]. In some cases, the concentration of swainsonine in the plants was comparable to the amount of the *A.* sect. *Undifilum* endophyte. Indeed, “SD mice” showed similar clinical and pathological symptoms after being fed with *A.* sect. *Undifilum* containing feed and as with dried *Oxytropis* plants [33].

This showed that the relationship between the *A.* sect. *Undifilum* endophyte and swainsonine is complex. For example, whilst *A.* sect. *Undifilum* was detected by qPCR in *Oxytropis* [54] or *Astragalus* samples [27], no detectable swainsonine was detected. Conversely, the endophyte could not be isolated from swainsonine-containing *O. latibracteata* Jurtz. samples (unpublished data). Additionally, *A.* sect. *Unidifilum* minus swainsonine producing *Oxytropis* samples could be reflecting the contributions by other fungal species. Swainsonine producing fungal species other that *A.* sect. *Undifilum* include *Metarhizium robertsii* (and *M. anisopliae*), *Slafractonia leguminicola* (previously known as *Rhizoctonia leguminicola*), and *Arthroderma otae* [60]. The above findings suggest that the biosynthesis of swainsonine in fungi is probably conserved in some lineage of symbiosis relationship.

Such observations naturally pose the question of the role of swainsonine for fungal species. In both plants and animals, the infectivity of fungi to their hosts could not be related to swainsonine production. In fact, swainsonine producing strains showed slightly less virulence than the knockout mutant. Inoculation experiments showed that by *M. robertsii* infection did not produce detectable swainsonine in maize (*Zea mays*), soybean (*Glycine max*), and milkvetch (*Astragalus sinicus*) [61]. Thus, the production of swainsonine is not necessary for colonization of those characterized fungi. It is worth noticing that although *Astragalus* and *Oxytropis* species are often colonized by *A.* sect. *Undifilum* and yield swainsonine, the infection of *M. robertsii*, which also produce swainsonine, did not trigger the swainsonine production in *A. sinicus*. This suggests that the fungi–host symbiosis to produce swainsonine are species specific, regardless of the diverse fungal lineage to produce the toxin.

### 1.4. The Biosynthesis Pathway of Swainsonine

**Figure 3:**
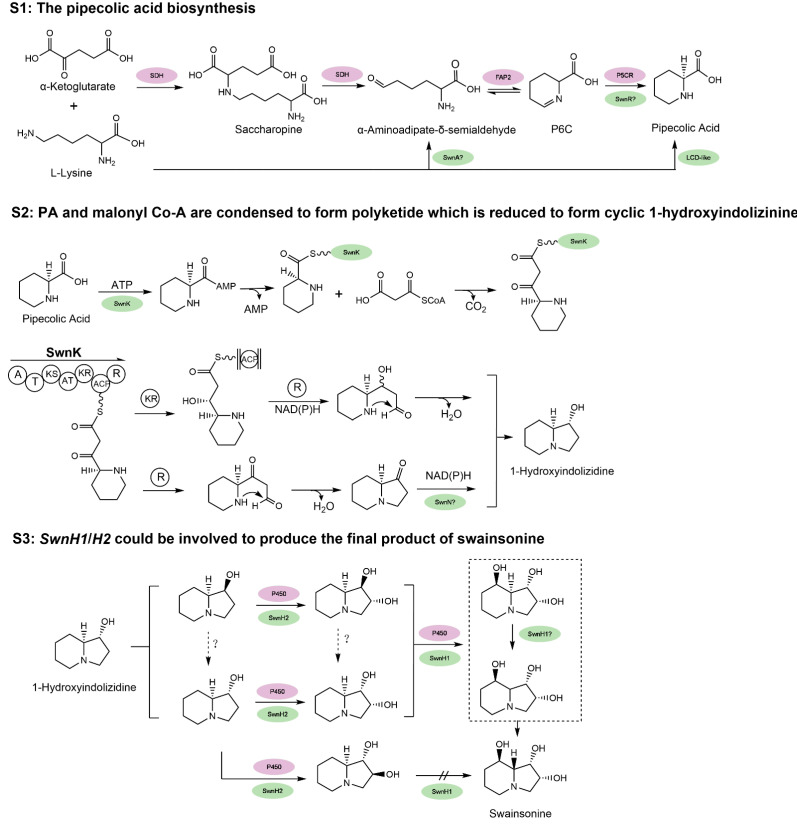
The hypothesized biosynthesis pathway of swainsonine. **S1**: The multiple proposed biosynthesis pathways of pipecolic acid (PA). **S2**: PA and malonyl Co-A are condensed to form polyketide which is reduced to form cyclic 1-hydroxyindolizinine. **S3**: 1-hydroxyindolizinine forms SW after redox reactions. For comparison, genes in purple indicate all the annotated genes with proposed function and position from the locoweed endophytic fungus *A. oxytropis*, based on its genome sequencing [63]; genes in green represent the proposed and some verified genes in *M. robertsii*, and “?” indicates that the function and position of activity of the gene still need further characterization [60,61]. Refer to the text for the name of the genes. Certain chemical structures and reactions in the schematics were adapted from previous work by Cook et al. and Luo et al. [60,61].

Despite its role as a locoweed toxin, the biosynthetic pathway of swainsonine has yet to be conclusively resolved. As swainsonine can be produced by several fungi that are not closely related phylogenetically, its biosynthetic pathway may have a deep evolutionary history. Based on previous work (see review by Tan et al. [62]), the pathway is arbitrarily divided into three stages (Figure 3). At stage 1 (S1), PA (pipecolic acid) is produced from lysine. At stage 2 (S2), PA and malonyl Co-A are condensed to form polyketide which is reduced to form cyclic 1-hydroxyindolizinine. Then, at stage 3 (S3), this direct precursor forms swainsonine after redox reactions. Several key steps in the biosynthesis pathway of swainsonine have been characterized in *S. legunimicola* and *M. anisopliae*. Additionally, a comparative genome sequencing revealed a multiple gene cluster, *SWN* and its orthologs as producing swainsonine [60]. The *SWN* cluster encodes seven putative genes, including *swnA* (an aromatic amino transferase gene), *swnH1* and *swnH2* (2-oxoglutarate- and Fe(II)-dependent dioxygenases gene), *swnN* (an NmrA-like, NADB Rossmann-fold reductase gene), *swnR* (an NADB Rossmann-fold reductase gene), *swnT* (a transmembrane transporter gene), and *swnK* (a multifunctional protein gene), which contribute to swainsonine production.

At S1, the first crucial precursor for swainsonine production is PA. In prokaryotes, PA can be synthesized at least by four possible routes [64]. Three of them employ LKR/SDH (lysine-ketoglutarate reductase/saccharopine dehydrogenase, not necessarily linked), LAT (lys aminotransferase), and LysDH (lysine dehydrogenase), respectively, to produce α-aminoadipate-δ-semialdehyde (AASA). AASA can spontaneously convert to P6C, which is further reduced by a P5CR (1-pyrroline-5 carboxylate reductase). The fourth path is AASA independent, which uses racemase, aminotransferase, and reductase to produce Δ1-piperidine-2-carboxylate (P2C) and then to PA. Currently it is not clear if such multiple pathways are all present in fungi. It was suggested that in yeast, lysine can be degraded at least by transaminase(s) and lysine dehydrogenase [65]. For swainsonine biosynthesis, Wickwire et al. [66] suggested PA is produced via the lysine → saccharopine → P6C (1-piperideine-6-carboylate) → PA but not the P2C route.

Some progress has been made to help understand PA formation during swainsonine biosynthesis. Continuing from the previous identification of the *SWN* cluster, Luo et al. [61] further studied its function in *M. robertsii*. The disruption of *swnA* decreased PA by five-fold and therefore it should play an important role in the production of PA. Since *swnA* was annotated as an aminotransferase, it may function similar to the bacteria LAT (Lys aminotransferase) in PA formation, which catalyze the direct transamination between L-lysine and α-ketoglutarate to form α-aminoadipate-δ-semialdehyde (AASA) and L-glutamate. However, this would skip the intermediate saccharopine suggested by Wickwire et al. [66]. *SwnR* and *SwnN* both encode reductase, and Luo et al. showed that in the knockout mutant of *swnR*, a considerable amount (~3/4) of PA was still produced compared with that of the WT. In the case of a *swnN* knockout, more PA was produced than the WT. This would suggest that *swnR* is more likely to be working during PA formation. It is not known if it encodes a P5CR like gene. Rather *swnN* would act after PA formation and its malfunction caused PA unable to be converted to downstream metabolites. Since the disruption of *swnA* or *swnR* could still produce PA, it was thus believed that another PA biosynthesis pathways exists [61]. An LCD (lysine cyclodeaminase)—similar to the RapL from the rapamycin gene cluster in *Streptomyces hygroscopicus* [67]—involved pathway was verified by that one copy of the two LCD genes in *M. robertsii* could produce substantial PA [61]. It is worth mentioning that *swnA* mutant almost abolished swainsonine production but given that PA was still there via a possible redundant route, we suggested that the route that *swnA* is involved is responsible for the main production of swainsonine and must also play a regulatory role to other downstream genes for swainsonine biosynthesis.

At S2, both Cook et al. [60] and Luo et al. [61] proved that interruption of the *swnK* gene resulted in no detectable swainsonine. The multifunctional *swnK* therefore plays a key role as a PKS (polyketide synthase) at S2 to form the first intermediate eight-carbon cyclic precursors containing a C3=N+4 iminium ion. Evidence from Luo et al. [61] suggested depending on whether the polyketide complex is reduced once (R) or twice (KR and R) by the swnK reductase domains, next, the formation of 1-hydroxyindolizine can be swnN dependent or independent. It is not clear how the C3=N+4 iminium ion could be reduced to form 1-hydroxyindolizidine. Since *swnN* significantly reduced swainsonine production it may work at this stage as suggested.

At S3, from 1-hydroxyindolizidine, SwnH2 and H1 may catalyze sequentially the two hydroxylation steps to produce the final product of swainsonine, as its production was abolished by the disruption of either of them. Since many isomeric intermediates and substrate specificity have been found during these steps, it remains unclear how exactly SwnH2 and H1 elaborate at S3. *SwnT*, as predicted, is indeed involved in mediating the secretion of SW. Since deletion of *swnA*, *swnK*, and *swn H1*&*2* made no detectable SW, they represent the key genes in SW biosynthesis. SwnN and swnR knockouts also yielded significant deceased amount of SW (10-fold and 5-fold, respectively), and thus should function in the main swainsonine pathway. The exact function and position of the several *SWN* genes remain to be elucidated.

Despite the progress in *S. legunimicola* and *M. robertsii*, the swainsonine biosynthesis pathway is unclear in *A.* sect. *Undifilum*. Experimental evidence is scarce. Mukerjee et al. [68] cloned a saccharopine reductase gene from *U. oxytropis*, and found the deletion mutant had decreased levels of saccharopine and lysine but increased level of PA and swainsonine. However, when Li and Lu [69] knocked out the homologous saccharopine reductase gene in *A. oxytropis* there were decreased levels of swainsonine and the levels of lysine were unaffected. This equivocal situation has yet to be resolved and, to date, no other genes have been clearly established in the proposed swainsonine pathway.

The genome of *A.* sect. *Undifilum* conspecies has been sequenced by two groups. Lu et al. [63] performed a ~56X depth sequencing using the Illumina Hiseq platform and assembled a 70.05 megabases (Mb) draft genome of a *U. oxytropis* isolated from China. Cook et al. [60] used an Illumina MiSeq platform to establish a ~78X fold depth of sequencing and reported an assembly of a 112.67 Mb *A. oxytropis* genome. The differences of genome size may be due to the two conspecies in *A.* sect. *Undifilum* represent different species/strains or heterozygosity in *A. oxytropis*. Lu et al. [63] defined five SDH (saccharopine dehydrogenase), one FAP2 (saccharopine oxidase), and two P5CR (pyrroline-5-carboxylate reductase) genes in the genome of *U. oxytropis*, as well as 77 PKS (polyketide synthase genes) and 79 P450 genes. The multiple SDH and P5CR genes Lu et al. [63] identified may represent different genes in the two family. However, given the large number of PKS and P450 genes, it is unlikely all of those genes represent different singletons specific to this pathway. It would be worthwhile to compare the synteny of those *SWN* genes as defined by Luo et al. [61] with genes previously annotated by Lu et al. [63], as many of the genes such as the SDHs and P450s have not been well characterized in previous work in *M. robertsii* and may share the similar function. It is worth noticing that the *swnA* and *swnT* genes are absent in the *A. oxytropis SWN* cluster [60]. The absence of *swnA* may reflect that either they have not been identified in the genome, or they were lost during evolution. As the deletion of *swnA* could result in little production of swainsonine in *M. robertsii*, it seems likely that other routes for PA accumulation readily exist in *A. oxytropis*. The loss of *swnT* may be related to the secretion of swainsonine as in this endophyte. The above requires further investigation. Li and Lu [69] compared the transcriptomes of WT and saccharopine-reductase-gene knockout *A. oxytropis* mutant to indicate differential expression by several genes related to the swainsonine biosynthesis. However, these genes were not annotated nor characterized making it difficult to summarize their function. A P2C (1-piperrideine-2-carboxylate) pathway was tentatively suggested by the authors to produce PA in *A. oxytropis* but this needs confirmation as previous work in *S. legunimicola* suggested otherwise [66]. Undoubtedly, better genome sequencing assemblies are required to begin to address this issue and group, including our own, are producing such data.

### 1.5. The Speculated Ecological Role of the Symbiosis in Oxytropis and Its Evolution

To date, the exact ecological relevance of the symbiosis between *Oxytropis* spp. and *A.* sect. *Undifilum* is unclear. This is in marked contrast to other mutualistic symbionts where endophytic interactions have demonstrated benefits to their host, for example in nutrient uptake and stress tolerance. In contrast, the benefits to *Oxytropis* through interaction with *A.* sect. *Undifilum* appear to be marginal at best [20]. It is clear that, unlike the case in poaceae, swainsonine cannot deter livestock grazing but may provide other cryptic benefits to the hosts. As we have discussed in the previous section, swainsonine is synthesized via the lysine → saccharopine (or skipped) → AASA→ P6C → PA → SW route in the fungi [62,66]. It may be relevant that this pathway is reminiscent of lysine catabolism in plants, and this could be a key observation in defining an ecological role for the *Oxytropis* spp. and *A.* sect. *Undifilum* interaction.

To explore the possible ecological role of lysine-PA-swainsonine metabolism in the symbiosis of *Oxytropis* spp. and *A.* sect. *Undifilum*, we speculated that it could be retributed to stress response. Lysine catabolism was suggested to be related to stress tolerance in prokaryotes [64]. Many genes in the catabolic pathway were up-regulated by osmotic stress. Similarly, lysine supplement increased the growth of *Silicibacter pomeroyito* high salinity, and expression of lysine catabolic operon increased salt tolerance and accumulation of more PA in *Escherichia coli*. Lysine catabolism was also suggested to be involved in plant stress tolerance [70]. Plant lysine catabolism is diverse, encompassing the cadaverine pathway, the SACPATH (saccharopine), and the NHP (*N*-hydroxypipecolate) pathways [64,71,72,73,74]. Under stress, levels of free lysine increase due to protein hydrolysis and, as a result several key enzymes in lysine transport and SACPATH pathways are induced in a lysine dependent manner [64]. These include such as the LKR/SDH and AASADH (aminoadipate semialdehyde dehydrogenase) and these many coupled to P5CR activities in order to keep the AASA concentrations to below toxic levels [75]. In the case of halophytes at least, this could be an adaptive pathway that sees increased levels of osmolytes proline and PA under salinity [47]. Being able to produce swainsonine from AASA, *A.* sect. *Undifilum* could also facilitate as an outlet to reduce the AASA. On the other hand, the NHP route seems to be deployed differently in making use of lysine to produce PA to induce systemic acquired resistance (SAR) following pathogen attack [76,77]. The endophyte may facilitate the host to produce PA from lysine when needed for downstream plant reaction. This would agree with previous findings that the abundance of *A.* sect. *Unidifilum* could regulate the assemblage of pathogens to the host [21,22]. Whether shared lysine catabolism is an ecological determinant of the interaction or not needs future work.

## 2. Future Perspectives

The understanding regarding host–endophyte interaction, irrespective of tremendous previous efforts, is still limited. This is particularly the case in *Oxytropis* spp. but the recent study in *Astragalus* the work by Harrison et al. [22] shed some light on the potential regulatory function of such symbiosis. Future research would possibly need to continue the focus on unravelling the mechanism in the process of symbiont establishment, model of interaction and co-evolution of the host-endophyte, new possibilities of transmission, and swainsonine biosynthesis. The information is still lacking in how the endophyte recognizes a narrow range of the hosts and elaborately evades host defense as no symptoms have been observed in the *Oxytropis* hosts infected by *A.* sect. *Undifilum*. This must indicate that co-evolution has been functioning through either environmental selection or genetic drift. What benefits, if any, do the endophyte supply to the hosts are still unclear although we speculated the endophyte may cryptically defend the hosts against certain biotic attack via sharing some metabolic pathway. Delicate genomes of both the host and the endophyte can serve for the reference in identifying key genes in the said interaction. The endophyte may also facilitate the growth of *Oxytropis* spp., especially by strengthening phosphorous uptake by altering either signaling transduction or root architecture, as phosphorous is the biggest limiting growth factor in land plants, and such beneficial symbiosis have been seen in root endophyte *Colletotrichum tofieldiae* confers plant fitness benefits that are phosphate status dependent [78].

Although indicated as vertically transmitted, whether *A.* sect. *Undifilum* can be propagated via horizontal transmission in the field, is yet unknown. A better understanding of the pollination process in *Oxytropis* spp. will also help explain how the genus has evolved into both toxic and non-toxic lineage, due to the maintenance and loss of symbiosis with *A.* sect. *Undifilum*. A global assessment of the phylogenetic relationship in both the hosts and endophyte in *Oxytropis* genus is also remarkably interesting. Especially, given the medicinal potential of swainsonine in the treatment of tumor, strains with high levels of swainsonine production can be selected from natural population or via genetic manipulation.

## Figures and Tables

**Table 1:** Species and distribution of poisonous *Oxytropis* spp. in the natural grasslands of the Western United States and China.

Region	Dominant Species	Typical Area	Reference
America (5 species)	*Oxytropis* *sericea*	Utah; Arizona; New Mexico; Oklahoma; Kansas; Montana; Wyoming; Idaho; North Dakota; Texas; Alaska	[26,27]
	*Oxytropis lambertii*	Utah; Arizona; New Mexico; Oklahoma; Kansas	
	*Oxytropis campestris* var. *dispar*	Montana; Alaska	
	*Oxytropis besseyi* var. *salmonensis*	Montana; Wyoming	
	*Oxytropis besseyi* var. *bessey*	Montana; Wyoming	
China (8 species)	*Oxytropis glabra*	Alasan Left Banner, Inner Mongolia; Akqi, Xinjiang; Hangjin Banner, Inner Mongolia	[28,29,30]
	*Oxytropis falcate*	Yushu, Qinghai;	
	*Oxytropis ochrocephala*	Haiyuan, Ningxia; Qilian, Qinghai	
	*Oxytropis kansuensis*	Tianzhu, Gansu; Zeku, Qinghai; Hainan, Qinghai	
	*Oxytropis glacialis*	Ali, Tibet	
	*Oxytropis sericopetata*	Qusum, Tibet	
	*Oxytropis deflexa*	Qilian, Qinghai	
	*Oxytropis hirta*	Shanxi (North); Gansu (East)	

**Table 2:** Formally recognized *Oxytropis A*. sect. *Undifilum* associations and their typical location.

Host Plants	Location	References
*Oxytropis sericea*	New Mexico (NM), USA	[19,32]
*Oxytropis sericea*	Colorado (CO), USA	[32,33]
*Oxytropis sericea*	Utah (UT), USA	[16,32,34,35]
*Oxytropis sericea*	Wyoming (WY), USA	[34]
*Oxytropis lambertii*	NM, USA	[27]
*Oxytropis lambertii*	UT, USA	[36]
*Oxytropis lambertii*	Arizona, USA	[36]
*Oxytropis kansuensis*	Gansu, China	[30,37]
*Oxytropis kansuensis*	Qinghai, China	[31]
*Oxytropis ochrocepala*	Ningxia, China	[37,38]
*Oxytropis ochrocephala*	Qinghai, China	[39]
*Oxytropis sericopetala*	Tibet, China	[30]
*Oxytropis glacialis*	Tibet, China	[30]
*Oxytropis glabra*	Inner Mongolia, China	[37,40,41]
